# Controlled synthesized of ternary Cu-Co-Ni-S sulfides nanoporous network structure on carbon fiber paper: a superior catalytic electrode for highly-sensitive glucose sensing

**DOI:** 10.1186/s12951-024-02635-w

**Published:** 2024-06-27

**Authors:** Yuanyuan Li, Yi Duan, Jiangtao Lin, Jinghan Liao, Chao Xu, Fangqin Xue, Yourong Duan

**Affiliations:** 1 State Key Laboratory of Systems Medicine for Cancer, Shanghai Cancer Institute, Renji Hospital, School of Medicine, Shanghai Jiao Tong University, Shanghai, 200032 China; 2 Department of Gastrointestinal Surgery, Shengli Clinical Medical College of Fujian Medical University, Fujian Provincial Hospital, No. 134 Dongjie, Fuzhou, China

**Keywords:** Cu-Co-Ni-S nanoporous network structure, Self-supporting catalytic electrode, Nonenzymatic glucose sensor

## Abstract

**Background:**

Efficient monitoring of glucose concentration in the human body necessitates the utilization of electrochemically active sensing materials in nonenzymatic glucose sensors. However, prevailing limitations such as intricate fabrication processes, lower sensitivity, and instability impede their practical application. Herein, ternary Cu-Co-Ni-S sulfides nanoporous network structure was synthesized on carbon fiber paper (CP) by an ultrafast, facile, and controllable technique through on-step cyclic voltammetry, serving as a superior self-supporting catalytic electrode for the high-performance glucose sensor.

**Results:**

The direct growth of free-standing Cu-Co-Ni-S on the interconnected three-dimensional (3D) network of CP boosted the active site of the composites, improved ion diffusion kinetics, and significantly promoted the electron transfer rate. The multiple oxidation states and synergistic effects among Co, Ni, Cu, and S further promoted glucose electrooxidation. The well-architected Cu-Co-Ni-S/CP presented exceptional electrocatalytic properties for glucose with satisfied linearity of a broad range from 0.3 to 16,000 μM and high sensitivity of 6829 μA mM^− 1^ cm^− 2^. Furthermore, the novel sensor demonstrated excellent selectivity and storage stability, which could successfully evaluate the glucose levels in human serum. Notably, the novel Cu-Co-Ni-S/CP showed favorable biocompatibility, proving its potential for in vivo glucose monitoring.

**Conclusion:**

The proposed 3D hierarchical morphology self-supported electrode sensor, which demonstrates appealing analysis behavior for glucose electrooxidation, holds great promise for the next generation of high-performance glucose sensors.

**Supplementary Information:**

The online version contains supplementary material available at 10.1186/s12951-024-02635-w.

## Introduction

Diabetes, a chronic metabolic disease, leads to various metabolic disorders associated with fluctuations in blood glucose levels within the human body, significantly impacting patient health outcomes, possibilities for extending lifespan, and overall quality of life [1]. The International Diabetes Federation (IDF) estimates that the number of deaths due to diabetes is projected to rise steadily to approximately 700 million by the 2040s [2]. For quick patient screening and medical monitoring, it is of great importance to develop highly responsive and specific rapid diagnostic glucose sensors in clinics [3, 4]. To this day, glucose sensors used in commercial applications primarily rely on electrooxidation facilitated by glucose oxidase [5]. However, limitations such as inadequate long-term stability and complex immobilization processes restrict the widespread application of enzyme glucose sensors [6]. Consequently, an urgent need arises to explore alternative approaches to designing nonenzymatic glucose sensors [7].

For the effective functioning of glucose sensors with high performance, the choice of electrode materials is crucial. Notably, nanomaterials possess numerous active sites and a significantly large specific surface area, making them highly promising candidates for nonenzymic glucose sensors [8]. Noble metals, such as gold and platinum, exhibit exceptional catalytic performance and biocompatibility, making them ideal catalysts for nonenzymatic glucose sensors. However, their susceptibility to chloride ion deactivation poses a challenge alongside their relatively high cost [9, 10]. Transition metal sulfides have garnered considerable attention in recent times due to their abundant sources, good stability, high electrical conductivity, and excellent catalytic properties [11]. The rapid advancement of nanotechnology has led to the extensive utilization of transition metal sulfide nanomaterials in various fields, including supercapacitors, solar cells, photodetectors, and electrochemical sensors [12–14]. Transition metal sulfides, such as NiS, CoS, and CuS, have been employed as cost-effective catalysts for electrochemical enzyme-free glucose sensors owing to their remarkable electrocatalytic activity. Singh et al. [15] synthesized a crystalline β-NiS thin film by atomic layer deposition to construct an ultrasensitive electrochemical glucose sensor. Wu et al. [16] fabricated white ear-shaped CoS nanomaterials using the hydrothermal method for fast and sensitive detection of glucose content in human serum. Zhu et al. [17] utilized Cu_2_O as a template to synthesize hollow CuS nanocube structures with large surface area of 57.84 m^2^ g^− 1^, which exhibited distinguished electrochemical catalytic activity towards glucose. However, monometallic materials exhibit a limited number of active sites and restricted electrochemical activity, thereby constraining their application in non-enzymatic glucose sensors [18]. Electrodes based on multimetallic materials demonstrate enhanced electrochemical performance attributed to the synergistic effect they offer.

Compared to monometallic sulfide nanomaterials, bimetallic sulfide nanomaterials such as CuCo_2_S_4_, CoNi_2_S_4_, and NiCo_2_S_4_ exhibit enhanced electron transfer efficiency attributed to their abundant reaction sites and synergistic effects of mixed valence cations. Consequently, they demonstrate superior catalytic performance in glucose oxidation. Xu et al. [19] synthesized CuCo_2_S_4_ nanosheets on flexible carbon fiber fabrics using the one-step hydrothermal method to construct a sensitive enzyme-free glucose sensor. Compared with monometallic sulfides, the CuCo_2_S_4_ nanosheet-modified electrode has a better catalytic effect on glucose oxidation, and it can directly detect glucose at lower potentials (+ 0.35 V) with higher sensitivity (3852.7 μA mM^− 1^ cm^− 2^). Dong et al. [20] successfully prepared CoNi_2_S_4_ nanosheets grown on reticulated nitrogen-doped carbon foam by calcination and hydrothermal processes for detecting glucose levels in human serum. Compared with monometallic sulfide nanomaterials, the sensing performance of the CoNi_2_S_4_-based electrode on glucose oxidation is significantly enhanced, and it shows an excellent linear relationship between glucose concentrations in the range from 0.5 to12.5 mM and 12.5 to 30 mM. Babu et al. [21] fabricated three-dimensional flower-like NiCo_2_S_4_ nanostructures on Ni-modified cellulose filter paper, constructing a novel electrochemical glucose sensor. Compared with CoS, the NiCo_2_S_4_-based sensor exhibits enhanced catalytic performance for glucose oxidation, featuring a wide linear detection range (0.5 μM to 6 mM) and lower detection limit (50 nM). However, the preparation procedure for bimetallic sulfide nanomaterials is intricate and laborious, leading to challenges in quality control during the preparation process and hindering clinical translation efforts.

The synthesis techniques commonly employed for fabricating transition metal sulfide nanomaterials encompass hydrothermal, template-assisted, chemical vapor deposition, and electrodeposition methods [22–25]. Among various synthetic methods, electrochemical approaches have gained significant attention due to their inherent advantages, including facile and mild experimental conditions, convenient operational procedures, and environmental compatibility. Electrodeposition techniques, such as cyclic voltammetry (CV), potentiometric step, and two-pulse deposition, are extensively employed in various electrochemical techniques [26]. Among these techniques, CV is a commonly preferred method employed for synthesizing nanomaterials based on transition metal sulfides. For example, Evariste et al. [27] constructed a high-performance asymmetric supercapacitor by electrodeposition of Mo-Ni-Co-S microspheres on Ni foam using CV method. Ahmed et al. [28] employed the CV method to electrodeposit Mn-Ni-Co-S nanosheets onto carbon cloth, utilizing them as battery materials for asymmetric supercapacitors.

In this work, we employed the cyclic voltammetry (CV) technique to fabricate Cu-Co-Ni-S nanoporous mesh structures on a highly conductive, appealing 3D hierarchical carbon fiber paper (CP), aiming to develop a novel and *sensitive* electrochemical glucose sensor. The direct in-suit synthesis of Cu-Co-Ni-S on 3D CP skeleton can function as a self-supporting and binder-free catalytic electrode, benefiting electronic/ionic conductivity. The hierarchical architecture of Cu-Co-Ni-S/CP can effectively enhance the availability of redox sites for glucose molecules, expedite electron transportation, and optimize ion diffusion kinetics. Furthermore, incorporating Cu, Co, Ni, and S elements synergistically enhances electron transfer efficiency. Consequently, the fabricated electrochemical sensor based on Cu-Co-Ni-S exhibits exceptional catalytic activity towards glucose oxidation with high sensitivity of 6829 μA mM^− 1^ cm^− 2^. Furthermore, it displays exceptional selectivity in the presence of diverse interferences and superior storage stability. Notably, the biocompatibility evaluation suggests that Cu-Co-Ni-S/CP holds great potential for in vivo glucose sensing.

## Experimental

### Instruments

The phase composition of Cu-Co-Ni-S/CP was analyzed by DLMAX-2200 X-ray diffraction instrument (XRD, Rigaku Corporation, Japan, Cu Kα-ray, scanning rate of 8^°^ min^− 1^, scanning angle of 10–90^°^). The surface element valence states of Cu-Co-Ni-S/CP were analyzed using K-Alpha X-ray photoelectron spectroscopy (XPS, Thermo VG-Scientific, Al Kα Radiation Source). Scanning electron microscope (SEM) was used to characterize Cu-Co-Ni-S/CP. In this experiment, three-electrode system was used: counter electrode (platinum electrode), reference electrode (saturated Ag/AgCl electrode), and working electrode (CP). All electrochemical experiments were performed on the CHI660C electrochemical workstation (Shanghai Chenhua Co., Ltd.).

### Chemicals

H_2_O_2_ (30%), NaCl, KH_2_PO_4_, KCl, uric acid (UA), ascorbic acid (AA), dmaltose (Mal), fructose (Fru), and acetaminophen (AP), dopamine (DA), sucrose (Suc) were purchased from Alfa Aesha (China) Chemical Co., Ltd. Glucose (Glu), glycine (Gly), ethanol, CuCl_2_·2H_2_O, CoCl_2_·6H_2_O, NiCl_2_·6H_2_O, SC(NH_2_)_2_, NaOH and HCl were all purchased from Sinopharm Chemical Reagent Co., Ltd. CP (20 × 20 cm^2^) was purchased from Toray (China) Co., Ltd. Calcein/PI Cell Viability Assay Kit, and CCK-8 reagent were purchased from Beyotime (China) Co., Ltd. The water used throughout the experiment was ultrapure water (The resistivity is greater than 18.2 MΩ cm^− 1^).

The cell lines utilized in this study were procured from the American Type Culture Collection (ATCC) and maintained in DMEM medium supplemented with 10% FBS and 1% penicillin/streptomycin at a temperature of 37 °C within a humidified incubator containing 5% CO_2_. The cells were cultured using tissue culture flasks measuring 25 cm^2^, with regular passaging every alternate day.

The C57BL/6 mice (18–22 g) were housed in suitable conditions. All animal experiments followed the guidelines for the Care and Use of Laboratory Animals and received approval from China Pharmaceutical University.

### Preparation of Cu-Co-Ni-S/CP

**Fig. 1:**
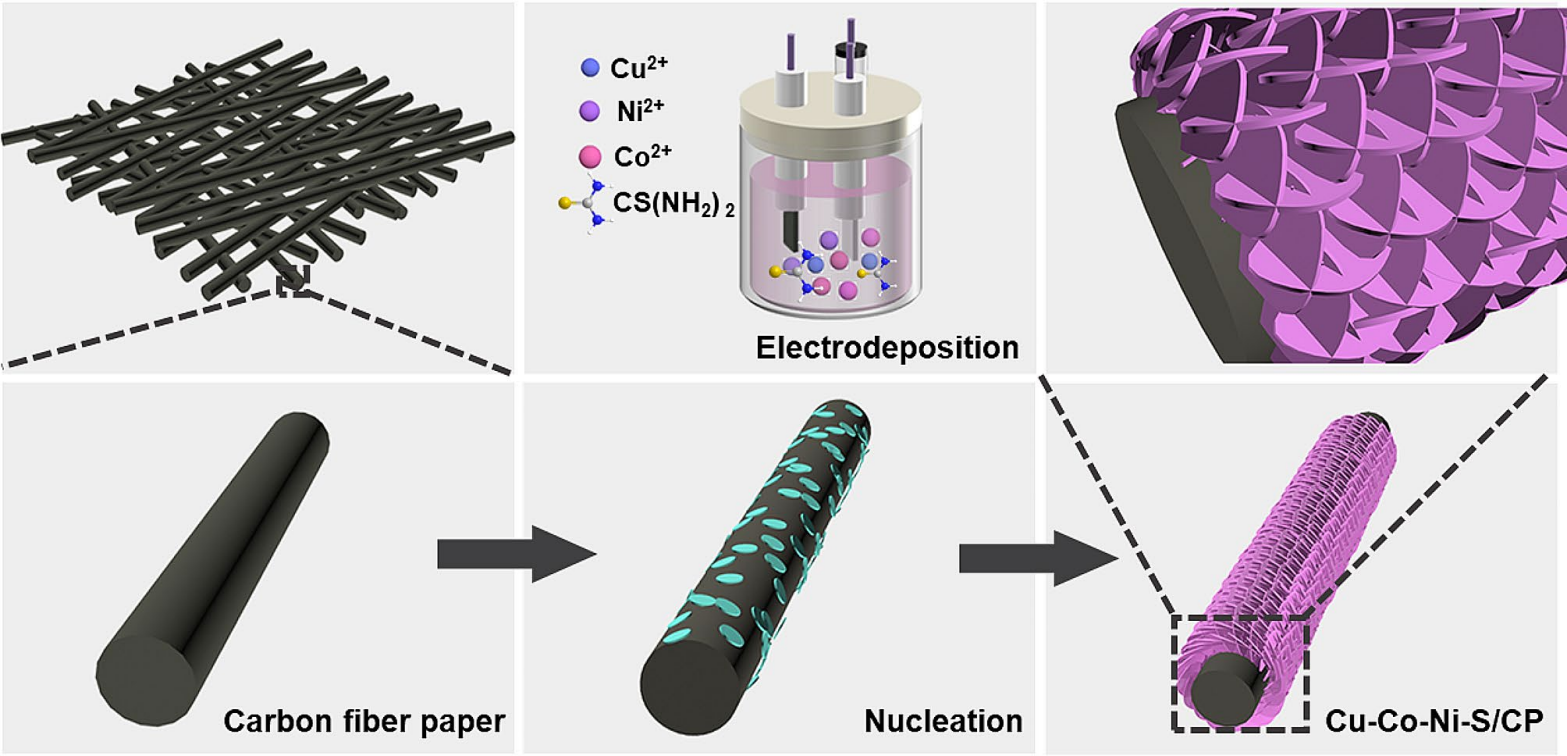
The growing process of Cu-Co-Ni alloy on CP by one-step electrodeposition

Figure 1 shows the synthesis of Cu-Co-Ni-S nanoporous network structure on CP. First, CP was pretreated to remove impurities on its surface. Then, CP was cut to the size of 2 cm × 2 cm and sonicated with HCl, ethanol, and water for 10 min in turn. Finally, CP was dried at room temperature. 5 mM CoCl_2_·2H_2_O, 7.5 mM NiCl_2_·2H_2_O, 75 mM SC(NH_2_)_2_, and CuCl_2_·2H_2_O solutions at different concentrations (0.5 mM, 1 mM, 2 mM, and 3 mM) were mixed to form electrodeposition solution. CV was used for electrodeposition in three-electrode system at sweep rate of 5 mV s^− 1^, deposition potential of − 1.2 V to + 0.2 V, and scan cycle of 3, 5, 7, and 9, respectively. For comparison, Ni-S/CP, Cu-Ni-S/CP, and Ni-Co-S/CP were prepared in the absence of CoCl_2_·6H_2_O and CuCl_2_·6H_2_O or CoCl_2_·6H_2_O in the electrodeposition liquid. The prepared material was fixed by insulated sealing film with an area of 0.25 × 0.25 cm^2^, connected to the electrode clamp as the working electrode to determine glucose in NaOH.

### Calcium and PI dye assays

HUVECs were cultured in 12-well plates overnight (1 × 105 cells per well) and subsequently treated with varying concentrations of Cu-Co-Ni-S/CP for a duration of 24 h. Following three washes with PBS, the cells were stained using 1 mL of a solution containing 2.0 μM PI (Beyotime, China) and 2.0 μM calcium indicator (Beyotime, China) for 30 min, then washed with PBS 3 times and imaged by microscopy (Olympus Corporation, Japan).

### In vitro cytotoxicity assay

The cytotoxic effects of the Cu-Co-Ni-S/CP on HUVECs were assessed employing the Cell Counting Kit-8 (CCK-8) assay (Beyotime, China). Cells were seeded into a 96-well plate overnight (5000 cells per well). Different concentrations of Cu-Co-Ni-S/CP were introduced at various incubation times. The control group consisted of the blank culture medium. Following that, the cells were subjected to an additional 24 h incubation period. Subsequently, the cytotoxicity was assessed using the CCK-8 assay in accordance with the guidelines provided by the manufacturer.

### In vitro hemolysis assay

10% red blood cell (RBC) suspension was prepared from C57BL/6 mice. Various concentrations of Cu-Co-Ni-S/CP were incubated with the RBC suspension for 1 h at 37 °C. The mixture was then centrifuged at 5000 rpm, 4 ℃ for 4 min. The resulting supernatant was collected for absorbance (OD) measurement at 540 nm using a microplate reader (Thermo, USA). H_2_O served as a positive control, and PBS was used as a negative control. The hemolysis rate was calculated: Hemolysis (%) = (OD_sample_ - OD_negative_ / (OD _positive_ - OD _negative_)) × 100%.

### In vivo evaluation of biocompatibility

All animal experiments were approved by the Animal Ethics Committee of the Shanghai Cancer Institute (License no. SYXK (Hu) 2012-0001). To assess the potential systemic toxicity of Cu-Co-Ni-S/CP, mice were euthanized after Cu-Co-Ni-S/CP or PBS treated for 24 h, and various organs, including heart, liver, spleen, lung, and kidney were collected for H&E staining.

### Detection of glucose in actual samples

The Cu-Co-Ni-S/CP electrode was utilized for the detection of glucose concentration in human serum through *the* standard addition method. Human serum samples were obtained from Shanghai Ruijin Hospital, which is affiliated with Shanghai Jiaotong University. To determine the glucose content, 5 μL of human serum was introduced into a solution containing 5 mL of NaOH (0.15 M).

## Results and discussion

### Morphological and characterization of Cu-Co-Ni-S/CP

Cu-Co-Ni-S/CP nanoporous network structure was obtained by one-step electrodeposition (Fig. 1). Electrodeposition liquids were composed of CoCl_2_·2H_2_O, NiCl_2_·2H_2_O, CuCl_2_·2H_2_O and CS(NH_2_)_2_. During electrodeposition, metal cations in the solution moved toward the cathode, nucleating on the surface of the cathode and growing to form the metal layer [29]. CoCl_2_·2H_2_O, NiCl_2_·2H_2_O, and CuCl_2_·2H_2_O were used as Cu-Co-Ni-S metal sources, while CS(NH_2_)_2_ was used as the sulfur source and metal ion complexing to allow Cu-Co-Ni-S to grow uniformly on the CP substrate. Thus, Cu-Co-Ni-S/CP nanoporous network structures were obtained at sweep velocities of 5 mV s^− 1^, deposition potentials of − 1.2 V to + 0.2 V, and 5 scan cycles.

**Fig. 2:**
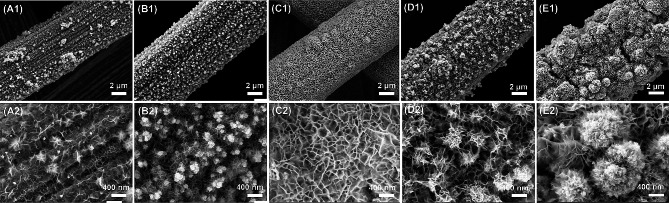
SEM images of Cu-Co-Ni-S/CP at different numbers of scan cycles: **A** 1. **B** 3. **C** 5. **D** 7. **E** 10

The topography of Cu-Co-Ni-S/CP was affected by the concentration of Cu^2+^ and number of scanned turns, so the topography of the sample was optimized by controlling the addition of Cu^2+^ concentration and the number of scanned laps. SEM was used to observe the microscopic topography of the samples. As shown in Fig. S1, the CP are composed of carbon fibers of approximately 6 μm diameter, with smooth surfaces. Fig. S2 showed the SEM plot of Cu-Co-Ni-S/CP at the addition of Cu^2+^ concentrations of 0.5 mM, 1 mM, 2 mM, and 3 mM, respectively. As shown in Fig. S2A and S2B, the structure of the nanoporous network was grown on the CP fiber substrate. With the increase of Cu^2+^ concentration (Fig. S2C–S2D), Cu-Co-Ni-S changed from the nanoporous network structure to the nanoparticle, and the nanoparticle became increasingly dense. This is because the concentration of Cu^2 +^ was too high, resulting in the rapid deposition of metal Cu. Therefore, Cu-Co-Ni-S tended to be granular, and the particles became more and more dense and gathered together [30]. When the concentration of Cu^2+^ was 1 mM, the nanoporous network structure of Cu-Co-Ni-S/CP was relatively uniform. Thus, we continued to change the number of scanning cycles (1, 3, 5, 7, and 9) when the Cu^2+^ concentration was 1 mM to optimize the morphology of the material. As shown in Fig. 2A, When the scanning cycle was 1, Cu, Ni, and Co ions tended to nucleate uniformly, leading to a thin layer consisting of fine nanosheets. When the scanning cycles were increased to 3, the adsorption of Cu, Ni, and Co ions on the CP substrate was accelerated, leading to the growth of uneven blocky particles on the surface of CP (Fig. 2B). When the scanning cycles were raised to 5, the nanoparticles grew longitudinally and then evolved into nanosheet arrays (Fig. 2C) [29, 31]. As the scanning cycles increased from 5 to 7, the architecture of Cu-Co-Ni-S changed from a uniform nanoporous network to an irregular hierarchical cobweb-like one (Fig. 2D). When the scanning cycles reached 10, the deposited composites were further stacked into blocks (Fig. 2E). Therefore, the Cu-Co-Ni-S/CP nanoporous network structure was obtained when the concentration of Cu^2+^ was 1 mM, the sweep rate was 5 mV s^− 1^, and the deposition potential was − 1.2 V to + 0.2 V. The number of scan turns was 5 turns.

**Fig. 3:**
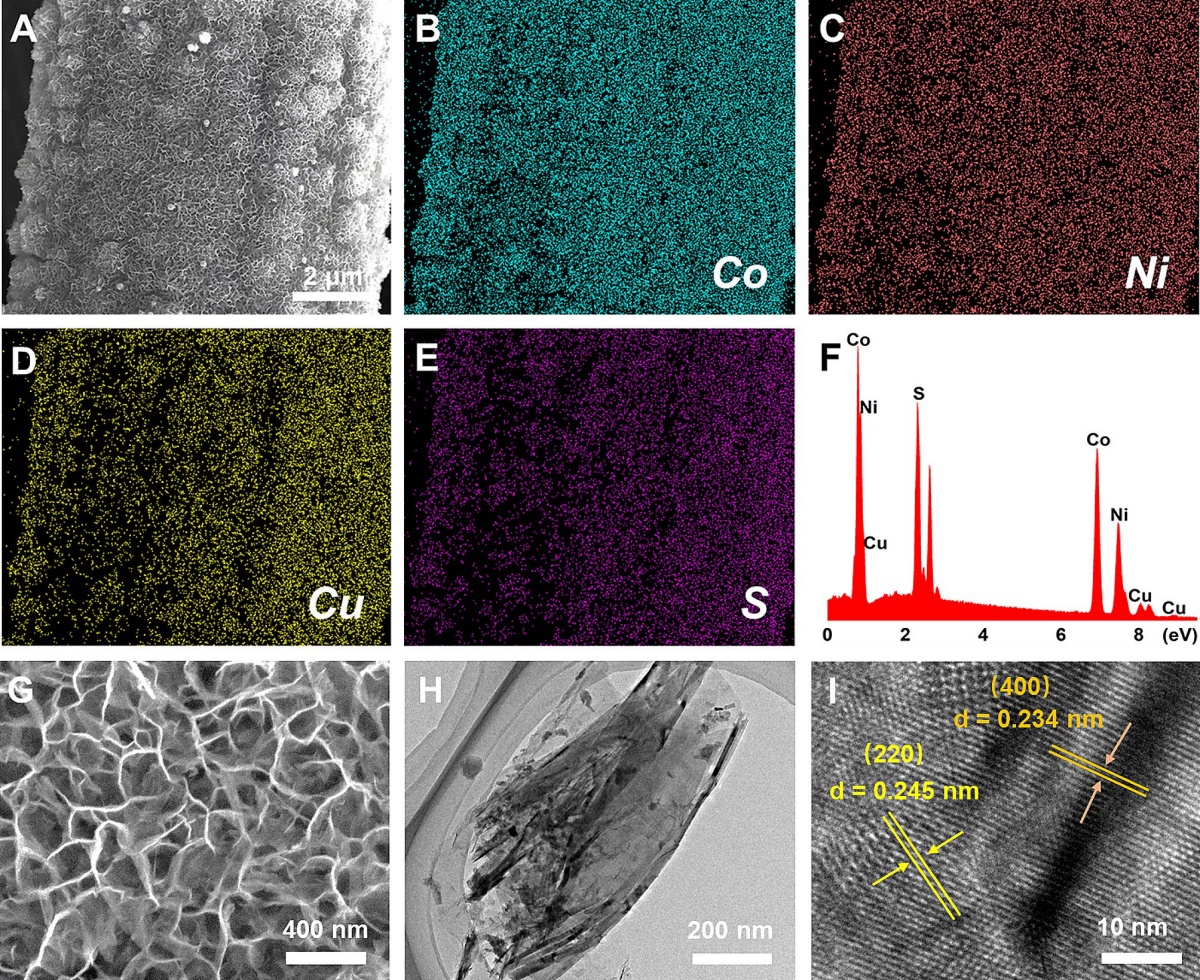
Characterizations of Cu-Co-Ni-S/CP nanostructure-based working electrode. **A** SEM image of Cu-Co-Ni-S/CP. **B**–**E** corresponding elemental mapping images of Co, Ni, Cu, and S. **F** EDS spectrum. **G** SEM image of the Cu-Co-Ni-S/CP in high magnification. **H** TEM image of Cu-Co-Ni-S/CP. **I** HRTEM image of Cu-Co-Ni-S/CP and insert is the selected area electron diffraction pattern

As shown in Fig. 3A, the relatively uniform Cu-Co-Ni-S nanoporous network structure was grown on the CP fiber substrate. The gap of the nanoporous network structure facilitates electrolyte entry and ion transport, increases the active site of the catalyst, accelerates electron transfer, and thus improves the electrocatalytic activity of the material for glucose oxidation. Figure 3B and E is the element mapping diagram of Cu-Co-Ni-S/CP, in which Cu, Co, Ni, and S elements were evenly distributed. Figure 3 F displayed the weight percentages of Co, Ni, Cu, and S elements accounting for 43%, 30%, 12%, and 15% of the total content, respectively. As shown in Fig. 3H, scaffold-like nanofoam was uniformly deposited on the surface of carbon fiber. The micromorphology of nanofoam was further analyzed by TEM, which revealed that nanofoam consisted of thin nanosheets (Fig. 3H). As shown in Fig. 2I, the high-resolution TEM (HRTEM) showed two lattice fringes with crystal plane distances of 0.234 nm and 0.245 nm corresponding to the (400) crystal plane of NiCo_2_S_4_ nanoparticles and the (220) crystal plane of CuS_2_, respectively. Additionally, the morphology of lower mixed phases for Cu-Ni-S and Co-Ni-S has been investigated. In comparison to the structure of Co-Ni-S/CP, a relatively compact porous structure of Cu-Ni-S film was observed on the carbon fiber surface, as depicted in Fig. S3. Furthermore, from the SEM image presented in Fig. S4, it can be seen that binary Co-Ni-S resulted in a rough surface structure composed of fine particles on the carbon fiber.

**Fig. 4:**
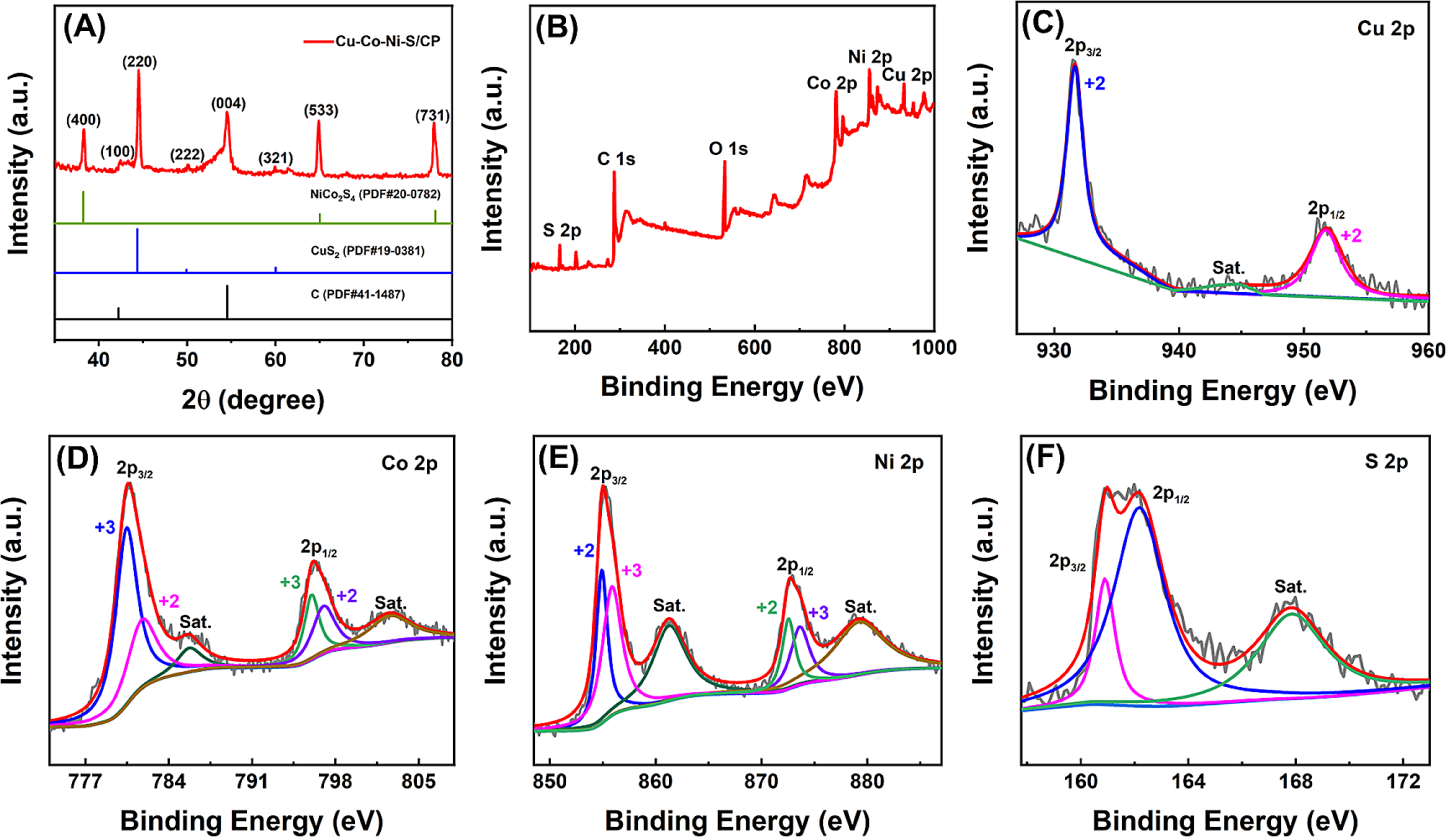
Characterizations of Cu-Co-Ni-S/CP nanostructure. **A** XRD patterns of Cu-Co-Ni-S/CP and Co-Ni-S/CP and XPS spectra of Cu-Co-Ni-S/CP. **B** Cu 2p. **C** Co 2p. **D** Ni 2p. and **E** S 2p

The crystal structure of the synthesized Cu-Ni-Co-S/CP was analyzed by XRD. As shown in Fig. 4A, the peaks that appeared at 42.22^°^ and 54.54^°^ corresponded to the (100) and (004) crystal planes (JCPDS card number 41-1487) in CP, respectively. The characteristic peaks at 44.37^°^, 49.78^°^, and 60.02^°^ corresponded to the (220), (222), and (321) crystal faces of CuS_2_ (JCPDS card number 19–0381), respectively [32]. The strong diffraction peaks that occurred at 38.31^°^, 65.08^°^, and 78.06^°^ correspond to the (400), (533), and (731) crystal faces of NiCo_2_S_4_ (JCPDS card number 20–0782), respectively [33].

The valence state and composition of the prepared Cu-Ni-Co-S/CP were further studied by XPS. As shown in Fig. 4B, Cu, Co, Ni, S, and C were the main elements on the surface of Cu-Co-Ni-S/CP. The binding energy of Cu^2+^ 2p_3/2_ and Cu^2+^ 2p_1/2_ peaks was 931.7 eV and 951.9 eV [34], respectively (Fig. 4C). In addition, the peak located around 943.8 eV was the satellite peak of Cu^2+^ 2p_3/2_. As shown in Fig. 4D, the two feature peaks at 780.6 eV and 782.0 eV corresponded to Co^3+^ 2p_3/2_ and Co^2+^ 2p_3/2,_ respectively, and the combined energy of the Co^3+^ 2p_1/2_ and Co^2+^ 2p_1/2_ peaks was 796.1 eV and 796.9 eV, respectively [33]. The two satellite peaks of Co^2+^ were located around 785.7 eV and around 802.5 eV. In the Ni 2p spectrum (Fig. 4E), the peaks at 855.0 eV and 872.7 eV belonged to Ni^2+^ 2p_3/2_ and Ni^2+^ 2p_1/2_ respectively, while the peaks at 856.0 eV and 873.7 eV corresponded to Ni^3+^ 2p_3/2_ and Ni^3+^ 2p_1/2_, respectively [33]. Moreover, the two peaks located at 861.3 eV and 879.4 eV were satellite peaks. In the XPS data of S 2p (Fig. 4F), the two feature peaks at 161.1 eV and 162.2 eV corresponded to 2p_3/2_ and 2p_1/2_ of S, respectively [35]. The XPS data provided additional verification that the electrodeposition process successfully facilitated the growth of a nanoporous network structure composed of Cu-Co-Ni-S on CP by one step.

**Fig. 5:**
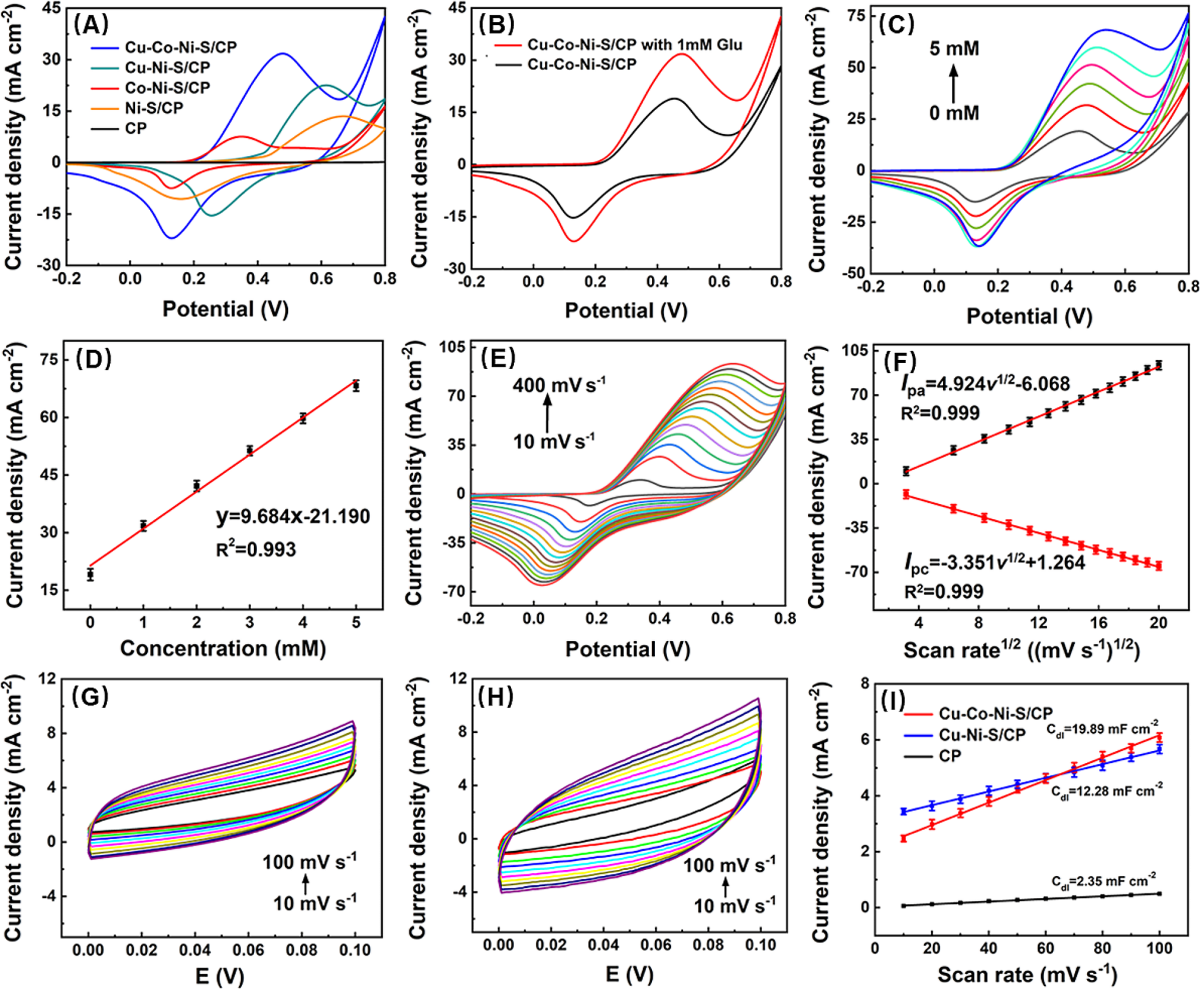
Electrocatalytic behavior of Cu-Co-Ni-S/CP for glucose detection. **A** CVs of the bare CP, Ni-S/CP, Co-Ni-S/CP, Cu-Ni-S/CP, and Cu-Co-Ni-S/CP in 0.15 M NaOH in the presence of 1 mM glucose. **B** CVs of Cu-Co-Ni-S/CP in the absence and presence of 1 mM glucose. **C** CVs of Cu-Co-Ni-S/CP with different concentrations of glucose in 0.15 M NaOH at the scan rate of 100 mV s^− 1^. **D** The calibration plot of the linear relationship between the oxidation peak current density and the concentration of glucose. **E** CVs of Cu-Co-Ni-S/CP in 0.15 M NaOH containing 1 mM glucose at different scan rates. **F** The calibration plot of the linear relationship between peak current density and the square root of scan rate. **G**, **H** CVs of Cu-Co-Ni-S/CP, Cu-Ni-S/CP electrodes measured in the applied potential range from 0.00 V to + 0.10 V at various scan rates. **I** The capacitive current density at + 0.05 V as the function of scan rate for Cu-Co-Ni-S/CP, Cu-Ni-S/CP and CP

### Electrocatalytic behavior of Cu-Co-Ni-S/CP for glucose

The electrocatalytic behavior of CP, Ni-S/CP, Cu-Ni-S/CP, and Cu-Co-Ni-S/CP on glucose was studied by CV. As shown in Fig. S5, with the addition of 1 mM glucose for Ni-S/CP, Cu-Ni-S/CP, and Co-Ni-S/CP electrode, generates different responses of peak current. Figure 5A shows the CV plots of CP, Ni-S /CP, Co-Ni-S/CP, Cu-Ni-S/CP, and Cu-Co-Ni-S/CP with 0.15 M NaOH at scanning speed of 100 mV s^−1^. The results showed that the CP electrode had no peak oxidation current after adding 1 mM glucose. Compared to Ni-S/CP, Co-Ni-S/CP, and Cu-Ni-S/CP, the oxidation current density observed on Cu-Co-Ni-S/CP in the presence of glucose was found to be significantly higher. The satisfactory behavior of Cu-Co-Ni-S/CP can be attributed to three factors: (a) Directly growing free-standing Cu-Co-Ni-S on a 3D CP skeleton avoids the use of binders and contributes to charge transfer; (b) The hierarchical nano/micro-architecture facilitates facile access of electrolyte ions, possesses abundant redox sites, and accelerates diffusion kinetics for glucose catalysis; (c) The synergistic coordination between Cu, Co, Ni, and S greatly increased the electrochemical sensing performance. Therefore, the Cu-Co-Ni-S/CP-based sensor shows excellent glucose oxidation capability.

As shown in Fig. 5B, the Cu-Co-Ni-S/CP electrode had significant oxidation peak current density at about 0.45 V, which may be the result of the combined action of Cu^3+^/Cu^2+^, Co^4+^/Co^3+^ and Ni^3+^/Ni^2+^ pairs of redox electron pairs during the electrooxidation of glucose. According to the XRD patterns, the main phases of Cu-Co-Ni-S/CP were composed of CuS_2_and NiCo_2_S_4_. Upon the addition of 1 mM glucose, an increase in oxidation current was observed along with a slight positive shift in the migration of the oxidation peak potential. During the anodic scan, Cu^2+^species on CuS_2_transformed into Cu^3+^[Eqs. 1], while Co^2+^and Ni^2+^species on NiCo_2_S_4_converted into Co^3+^and Ni^3+^due to an oxidation reaction [Eqs. (2 − 4)]. Subsequently, glucose molecules were rapidly oxidized to gluconolactone [32, 36]. The sensing mechanism of Cu-Co-Ni-S/CP towards glucose can be described as follows: during the anodic scan, Cu^2+^, Ni^2+^, and Co^3+^species present on the Cu-Co-Ni-S/CP are converted into Cu^3+^, Ni^3+^, and Co^4+^through an oxidation reaction, which facilitates rapid glucose oxidation to gluconolactone in its presence [Eq. (5)]:

1 $${\rm{Cu}}{{\rm{S}}_{\rm{2}}}{\rm{ + O}}{{\rm{H}}^ - } \to {\rm{Cu}}{{\rm{S}}_{\rm{2}}}{\rm{OH + }}{{\rm{e}}^ - }$$

2 $${\rm{CoS + O}}{{\rm{H}}^ - } \to {\rm{ CoSOH + }}{{\rm{e}}^ - }$$

3 $${\rm{CoSOH + O}}{{\rm{H}}^ - } \to {\rm{ CoSO + }}{{\rm{H}}_{\rm{2}}}{\rm{O + }}{{\rm{e}}^ - }$$

4 $${\rm{NiS + O}}{{\rm{H}}^ - } \to {\rm{ NiSOH + }}{{\rm{e}}^ - }$$

5 $$\begin{array}{l}{\rm{C}}{{\rm{u}}^{{\rm{3 + }}}}{\rm{ + C}}{{\rm{o}}^{{\rm{4 + }}}}{\rm{ + N}}{{\rm{i}}^{{\rm{3 + }}}}{\rm{ + glucose }} \to \\{\rm{C}}{{\rm{u}}^{{\rm{2 + }}}}{\rm{ + C}}{{\rm{o}}^{{\rm{3 + }}}}{\rm{ + N}}{{\rm{i}}^{{\rm{2 + }}}}{\rm{ + glucolactone}}\end{array}$$

As shown in Fig. 5C, as the concentration of glucose increased, there was a corresponding increase in the oxidation current density observed for Cu-Co-Ni-S/CP on glucose, indicating that Cu-Co-Ni-S/CP suggested excellent electrooxidation capability for glucose in alkaline solutions. Figure 5D showed that the oxidation current density and glucose concentration were in a good linear relationship in the range of 0 to 5 mM, and the linear equation is I (mA cm^− 2^) = − 21.190 + 9.684 C (mM), R^2^ = 0.993. Figure 5E shows the CV of Cu-Co-Ni-S/CP in a solution containing 0.15 M NaOH and 1 mM glucose at different scanning rates. When the scanning rate increased from 10 mV s^− 1^ to 400 mV s^− 1^, the oxidation peak current density and reduction peak current density also continued to increase. Furthermore, as the scanning rate was elevated, there was an observed shift toward positive and negative directions in the potentials of both the oxidation peak and reduction peak. This phenomenon can be ascribed to the generation of intermediate compounds on the surface of the active site electrode upon *the* addition of glucose to the electrolyte, impeding timely diffusion of glucose from the solution to the sensing electrode surface [20]. As shown in Fig. 5F, there exists a good linear correlation between the square root of the sweeping speed and both the oxidation current density and reduction current density of glucose. The linear equations were I_p.a._ (mA cm^− 2^) = − 6.068 + 4.924*v*^1/2^ (mV s^−1^)^1/2^ (R^2^ = 0.999) and I_pc_ (mA cm^− 2^) = 1.264 − 3.351 *v*^1/2^ (mV s^− 1^)^1/2^ (R^2^ = 0.999), respectively. This suggested that the glucose oxidation process of Cu-Co-Ni-S/CP is a diffusion control process [37].

**Fig. 6:**
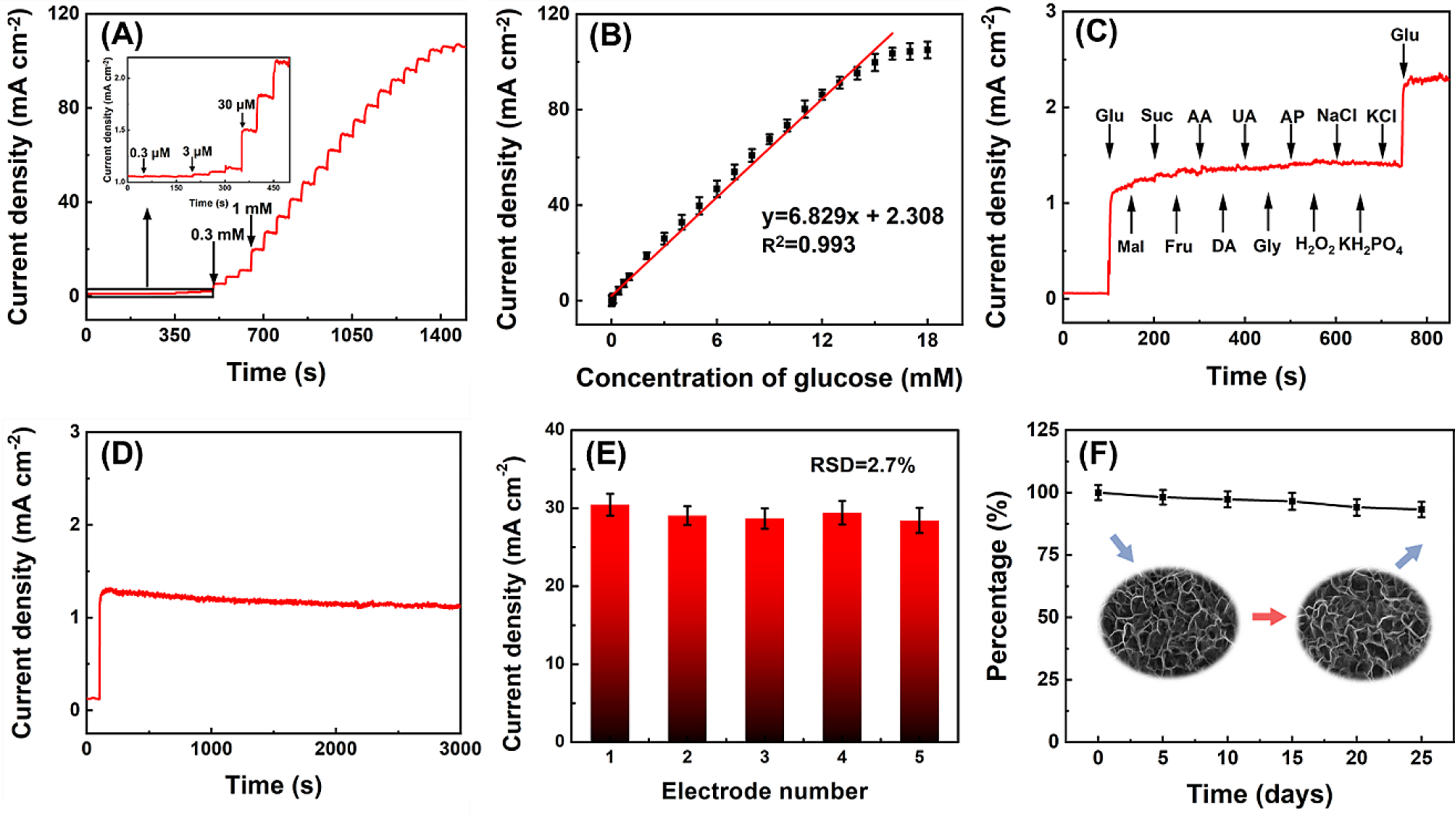
Electrochemical analysis performance of Cu-Co-Ni-S/CP for glucose detection. **A** Current-time curves obtained at Cu-Co-Ni-S/CP upon different concentrations of glucose in 0.15 M NaOH at + 0.45 V. **B** Relationship between the amperometric responses and the glucose concentrations. **C** Cu-Co-Ni-S/CP to 0.1 mM glucose and 0.01 mM different interferences. **D** The current density changes of Cu-Co-Ni-S/CP to 0.1 mM glucose during 3000 s test period. **E** The reproducibility test of Cu-Co-Ni-S/CP. **F** The storage stability of the Cu-Co-Ni-S/CP electrode. The insert showed SEM images of the electrode before and after electrochemical detection of glucose

The electrochemically active surface area value, which is directly proportional to the double-layer capacitance (C_dl_), can be utilized to determine the effective surface area of the electrode. The C_dl_ value is calculated by determining the slope of the linear regression equation relating sweep velocity to current density, and then halving the slope value [38]. We measured the electrochemically active surface area by the CV method. Figure 5G and H, and S6 were CV plots of Cu-Co-Ni-S/CP, Cu-Ni-S/CP, and CP, respectively. As shown in Fig. 4I, the C_dl_ values of Cu-Co-Ni-S/CP, Cu-Ni-S/CP, and CP are 19.89 mF cm^− 2^, 12.28 mF cm^− 2^, and 2.35 mF cm^− 2^, respectively. The Cu-Co-Ni-S/CP exhibited the highest C_dl_ value, approximately 1.6 times that of Cu-Ni-S/CP and around 8 times greater than CP, indicating a significantly larger electrochemical active surface area compared to Cu-Ni-S/CP. Therefore, Cu-Co-Ni-S/CP exhibited exceptional catalytic performance, surpassing that of both Cu-Ni-S/CP and CP.

### Study on the electrochemical analysis performance of Cu-Co-Ni-S/CP for glucose

To enhance the electrocatalytic efficiency of Cu-Co-Ni-S/CP towards glucose, the two experimental conditions of applied voltage and electrolyte concentration were optimized. As shown in Fig. S7A, it is evident that there was a positive correlation between the applied potential and the oxidation current density of glucose within the range of + 0.35 V to + 0.45 V. When the voltage surpassed + 0.45 V, a decline in oxidation current density was observed. Consequently, + 0.45 V was determined as the applied voltage. The electrolyte concentration also affected the electrochemical properties of the sensor. As shown in Fig. S7B, when the concentration of NaOH solution ranged from 0.05 M to 0.15 M, a significant increase in glucose current density response on Cu-Co-Ni-S/CP was observed. However, upon exceeding the NaOH concentration of 0.15 M, the oxidation current density of glucose decreased. The reason for this phenomenon can be explained that OH^−^ is conductive to the oxidation of glucose, but too high concentration of OH^−^ may lead to oxygen evolution reaction, thereby blocking the active site on Cu-Co-Ni-S/CP and thus hindering its catalysis of glucose oxidation [39]. Therefore, in subsequent experiments, 0.15 M NaOH was chosen as the experimental electrolyte.

Under optimal experimental conditions, the current-time technique was employed to investigate the response of Cu-Co-Ni-S/CP in 0.15 M NaOH solution towards varying glucose concentrations. The standard curve in Fig. 6A shows that the *response* currents of glucose by Cu-Co-Ni-S/CP increased linearly over the range of 0.3 μM to 16 mM concentrations. As shown in Fig. 6B, the linear equation is I (mA cm^− 2^) = 2.308 + 6.829 C (mM) (R^2^ = 0.993) with sensitivity and detection limits of 6829 μA mM^− 1^ cm^− 2^ and 0.1 μM (*S*/*N* = 3), respectively. As shown in Table 1, compared with the performance of other glucose sensors previously reported in the literature, the objective sensor had broader linear range, lower detection limit, and higher sensitivity, indicating that Cu-Co-Ni-S/CP with unique morphology and structure exhibited satisfactory catalytic performance for glucose electrooxidation.

**Table 1:** Comparison of properties between Cu-Co-Ni-S/CP and other glucose sensors

Modified electrode	Linear range (μM)	Detection limit (μM)	Sensitivity (μA mM^− 1^ cm^− 2^)	Reference
Ni_3_S_2_/NCNT^a^	0.46–3190	0.14	1447.64	[40]
Ni-MOF^b^/NiS_*1.03*_	2–1320	0.2	969	[41]
Cu_2_O@Cu_1.8_S/GC^c^	10–3100	1.30	61.67	[42]
Co_3_S_4_/GCE^d^	2–1110	0.17	346.70	[43]
CoS@Co-MOF/GCE	5–1170	0.11	4.6	[44]
CoNi_2_S_4_/NWE^e^	1–8000	0.30	2473	[45]
CuS/CoS	100–11,000	1.71	314.85	[46]
Cu_x_S-MoS_2_-rGO^f^/GCE	2–6330	0.60	308.20	[47]
Co-CuS/GCE	1–3660	0.1	1475.97	[48]
CoNiS/CF^*g*^	5–3470	2.0	2298.7	[49]
P^h^-NiCo_2_S_4_	1–5200	0.46	250	[50]
Cu-Co-Ni-S/CP	0.3–16,000	0.10	6829	This work

Notes: ^a^ nitrogen-doped carbon nanotubes, ^b^ metal-organic framework, ^c^ glassy carbon disc, ^d^ glassy carbon electrode ^e^ Ni wire electrode, ^f^ reduced graphene oxide, ^g^ Cu foam, ^h^ phosphorus doping

The current-time technique was employed to study the selectivity of Cu-Co-Ni-S/CP. Carbohydrates such as Fru, Suc, and Mal, as well as other substances such as UA, AA, Gly, DA, H_2_O_2_, AP, NaCl, KH_2_PO_4_, and KCl, were commonly associated with glucose in human serum, but the glucose content was exceeded that of other interfering substances by approximately 30 times [51]. In a solution containing 0.15 M NaOH, glucose was initially introduced at a concentration of 0.1 mM, followed by the sequential addition of UA, AA, Gly, DA, Fru, Suc, Mal, AP, H_2_O_2_, NaCl, KH_2_PO_4_, and KCl at concentrations of 0.01 mM. The concentration of added glucose, although 10 times higher than that of other interfering substances, remains significantly lower compared to the concentration of glucose in actual human serum samples. Therefore, investigating the selectivity of the sensor holds substantial significance. As depicted in Fig. 6C, the response current density produced by the interfering substance was much smaller than that of glucose, which indicated that the sensor had good selectivity.

To assess the stability and reproducibility of the prepared sensor, 0.1 mM glucose is added to the 0.15 M NaOH solution to evaluate the sensing ability of the Cu-Co-Ni-S/CP. The stability of the sensor can be observed from the consistent trend shown in Fig. 6D over a duration of 3000 s. As shown in Fig. 6E, five Cu-Co-Ni-S/CP electrodes were fabricated using identical experimental parameters, and the relative standard deviation (RSD) calculated from their detection results was 2.7%, indicating that the prepared sensor had good reproducibility. As shown in Fig. S8, the same electrode was repeated 20 times in 0.15 M NaOH solution containing 1 mM glucose, and the RSD calculated from their test results was 1.8%, indicating that the glucose sensor had good reusability. To investigate the storage stability of the sensor, the 0.15 M NaOH solution containing 1 mM glucose was detected every 5 days by the same electrode, and the response of Cu-Co-Ni-S/CP to the density of glucose oxidation current was observed. As shown in Fig. 6F, the response current drops to 93.22% of the beginning value after 25 days, suggesting that the Cu-Co-Ni-S/CP electrode had good storage stability. Additionally, the unique architecture of the electrocatalyst remained intact even after a prolonged period of 25 days (inserted in Fig. 6F), with negligible alterations in material morphology observed, thereby indicating exceptional structural durability. The slight decrease in the current response may be due to a small amount of passivation on the electrode surface. The current response of glucose oxidation exhibited a slight decline, which could potentially be attributed to a negligible passivation phenomenon occurring at the surface of the electrode.

**Fig. 7:**
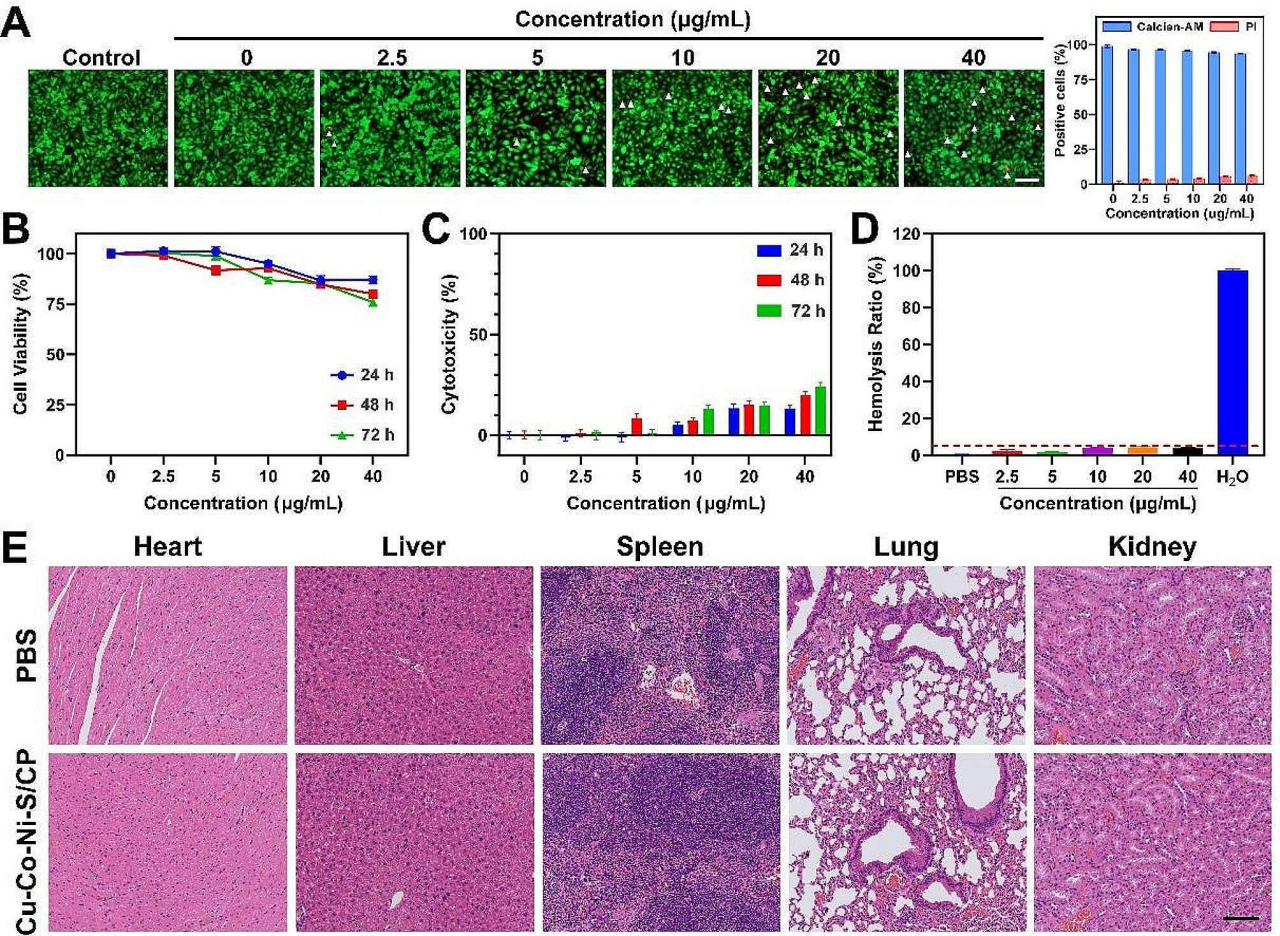
Evaluation of biocompatibility behaviors of Cu-Co-Ni-S/CP. **A **Calcein-AM and PI staining of HUVECs treated with various concentrations of Cu-Co-Ni-S/CP for 24 h and the quantitative analysis (*n* = 3 independent samples). White triangles indicate the apoptotic cells. Scale bar = 100 μm. **B, C **Cell viability and cytotoxicity analysis of HUVECs treated with various concentrations of Cu-Co-Ni-S/CP for 24, 48, and 72 h (mean ± SD, *n* = 3 independent samples). **D** Hemolysis test of Cu-Co-Ni-S/CP treated with various concentrations of Cu-Co-Ni-S/CP for 2 h. **E** Histological microscopic images stained with H&E. Scale bar = 100 μm

### The biosafety of Cu-Co-Ni-S/CP

Although Cu-Co-Ni-S/CP exhibits excellent performance for glucose electrooxidation, the biocompatibility of Cu-Co-Ni-S/CP should be explored for further in vitro and in vivo applications. To assess the cytotoxicity of Cu-Co-Ni-S/CP at different concentrations, live/dead staining was conducted. The results depicted in Fig. 7A indicate that when administered for 24 h, Cu-Co-Ni-S/CP had minimal impact on the viability of HUVECs at concentrations equal to or below 20 μg mL^− 1^. However, a semi-quantitative examination of the live/dead staining demonstrated an elevation in PI-positive cells and a reduction in Calcein-AM-positive cells when exposed to concentrations of 1 and 40 μg mL^− 1^. Even at concentrations as high as 40 μg mL^− 1^, the increase in the proportion of dead cells was minimal. To further explore the impact of Cu-Co-Ni-S/CP on the viability of HUVECs, a CCK-8 assay was conducted following the administration of Cu-Co-Ni-S/CP for 24, 48, and 72 h. The findings revealed that at a concentration of 20 μg mL^− 1^, Cu-Co-Ni-S/CP exhibited minimal cytotoxicity towards HUVECs. However, as the concentration increased to 40 μg mL^− 1^, the decrease in cell viability was observed to be dependent on the dosage and duration of exposure (Fig. 7B, C). In addition, the hemocompatibility of Cu-Co-Ni-S/CP was evaluated using a hemolysis test to examine its impact on blood cell homeostasis at the nanoscale. The results depicted in Fig. 7D demonstrate that even at a concentration of 40 μg mL^− 1^, Cu-Co-Ni-S/CP had minimal detrimental effects on the viability of RBCs, with a hemolysis rate below 3%. These findings collectively suggest that Cu-Co-Ni-S/CP exhibits satisfactory biocompatibility when used at or below a dose of 40 μg mL^− 1^ in HUVECs. Moreover, following the sacrifice of the mice, major organs were obtained and subjected to H&E staining for analysis (Fig. 7E). Notably, no apparent tissue damage was observed, indicating the favorable biosafety of Cu-Co-Ni-S/CP. The above results suggest that our developed electrochemical sensor holds significant potential for in vivo blood glucose levels detecting.

**Table 2:** Determination results of glucose in human serum sample

Sample	Detected (mM)	Commercial method (mM)	Recovery (%)	RSD (*n* = 3) (%)
1	4.52	4.59	99.40	2.6
2	4.83	4.72	101.40	1.5
3	7.69	7.60	100.20	2.3
4	7.88	7.10	99.02	2.4
5	6.10	6.52	98.90	1.3
6	8.83	8.10	99.89	1.8

**Fig. 8:**
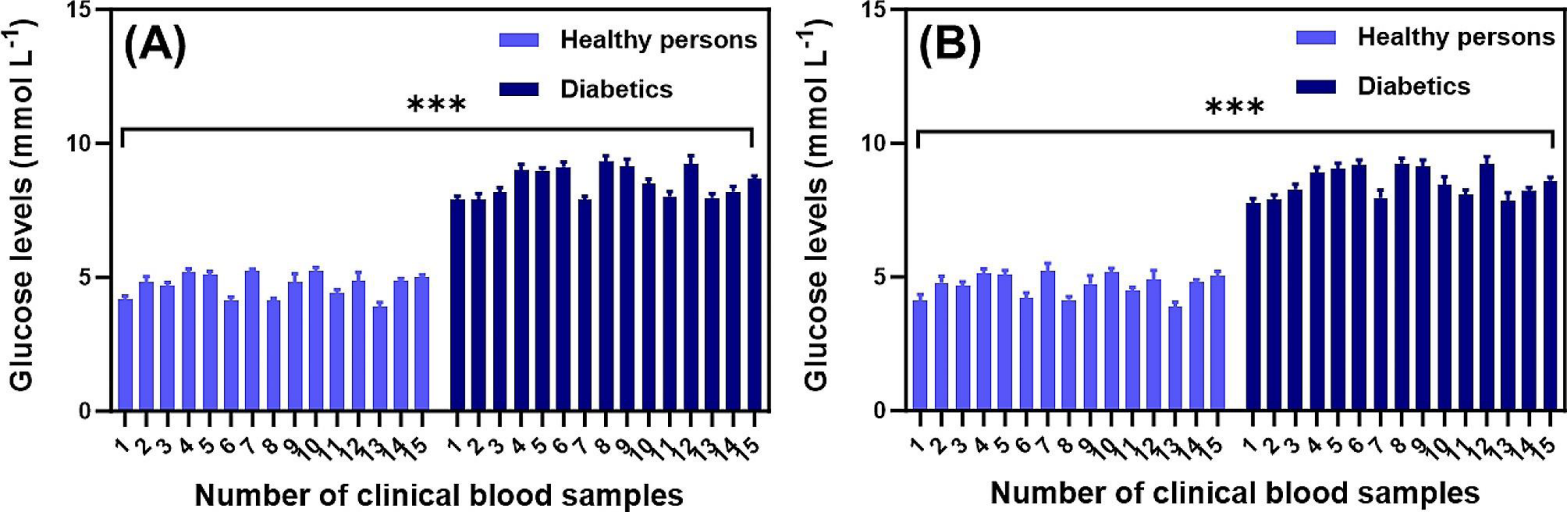
Profiling glucose levels of human blood samples. Analysis of glucose levels by **A** Cu-Co-Ni-S/CP and **B** blood glucometer in clinical human of health and patient blood samples. Error bars represent mean ± SD of 3–5 independent experiments. Differences with ***P < 0.0001 were considered statistically significant by *t* test

### Determination of glucose in human serum samples

To assess the effectiveness of Cu-Co-Ni-S/CP, we employed the standard addition technique to quantify glucose levels in human serum. Additionally, we compared the results obtained from commercial methods for measuring glucose concentrations in human serum samples, which were evaluated using a HEA-215 blood glucose meter. The HEA-215 blood glucose meter, manufactured by Omron Co., Ltd. in China, is a commercially available device for measuring blood glucose levels. As shown in Table 2, the recovery rate was 98.90 to 101.40%, and the RSD was 1.3 to 2.6%, which was closely aligned with the commercial method. To further demonstrate the practical application of Cu-Co-Ni-S/CP, a total of thirty clinical blood samples were obtained for analysis. Specifically, fifteen samples were sourced from healthy individuals, while the remaining fifteen samples were collected from patients diagnosed with diabetes. As shown in Fig. 8A, the electrochemical sensor we prepared can accurately detect blood glucose levels in clinical blood of both healthy people and diabetic patients. Additionally, its detection results are consistent with those of blood glucometer (Fig. 8B). The above findings demonstrate the successful development of a highly precise sensor capable of accurately monitoring blood glucose levels in clinical samples, enabling reliable differentiation between healthy individuals and those with diabetes. Thus, the prepared glucose sensor holds significant potential for clinical applications.

## Conclusions

In summary, the Cu-Co-Ni-S/CP electrode was prepared for the first time by simple one-step electrodeposition. The nanoporous network structure of Cu-Co-Ni-S significantly increases the active site of the material and accelerates electron transfer. In addition, the synergy between Cu, Co, Ni, and S and the 3D network structure composed of CP increase the conductivity of the nanomaterials, thereby improving the electrocatalytic performance of Cu-Co-Ni-S/CP. Based on the above advantages, the prepared Cu-Co-Ni-S/CP electrode has a wide linear range (0.3 μM to 16 mM), low detection limit (0.1 μM), high sensitivity (6829 μA mM^− 1^ cm^− 2^), and good anti-interference and stability. The Cu-Co-Ni-S/CP electrode was applied to the detection of glucose in human serum, and a satisfactory recovery rate was obtained. Furthermore, the Cu-Co-Ni-S/CP displayed good biocompatibility, suggesting that the sensor had potential application value in clinic.

### Electronic supplementary material

Below is the link to the electronic supplementary material.

Supplementary Material 1

## Data Availability

No datasets were generated or analysed during the current study.
