# Supplementary material for: Controlled synthesized of ternary Cu-Co-Ni-S sulfides nanoporous network structure on carbon fiber paper: a superior catalytic electrode for highly-sensitive glucose sensing
Source: J Nanobiotechnology. 2024 Jun 27;22:377. doi: 10.1186/s12951-024-02635-w (PMC11210160; doi:10.1186/s12951-024-02635-w)
Supplement: Supplementary file 1 — Supplementary Material 1 [file 12951_2024_2635_MOESM1_ESM.docx]

**Controlled Synthesized of** **Ternary Cu-Co-Ni-S** **Sulfides Nanoporous Network Structure on Carbon Fiber Paper: A Superior Catalytic Electrode for Highly-Sensitive Glucose Sensing**

Yuanyuan Li^1^, Yi Duan^1^, Jiangtao Lin^1^, Jinghan Liao^1^, Chao Xu^2^, Fangqin Xue^2,*^, Yourong Duan^1,*^

^1^State Key Laboratory of Systems Medicine for Cancer, Shanghai Cancer Institute, Renji Hospital, School of Medicine, Shanghai Jiao Tong University, Shanghai 200032, China.

^2^Department of Gastrointestinal Surgery, Shengli Clinical Medical College of Fujian Medical University, Fujian Provincial Hospital, No. 134 Dongjie, Fuzhou, China.

^*^Corresponding authors:

Correspondence to Yourong Duan (E-mail: [yrduan@shsci.org](mailto:yrduan@shsci.org)) and Fangqin Xue (E-mail: [xuefangqingsl@sina.com](mailto:xuefangqingsl@sina.com))

**
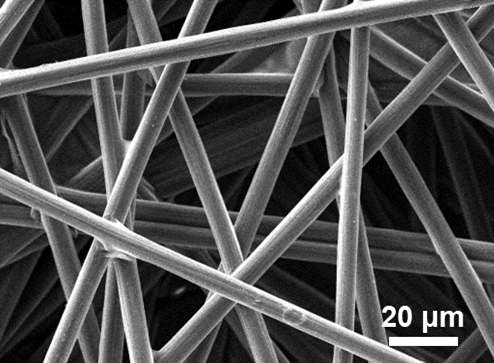
**

**Fig. S1.** SEM image of bare CP.


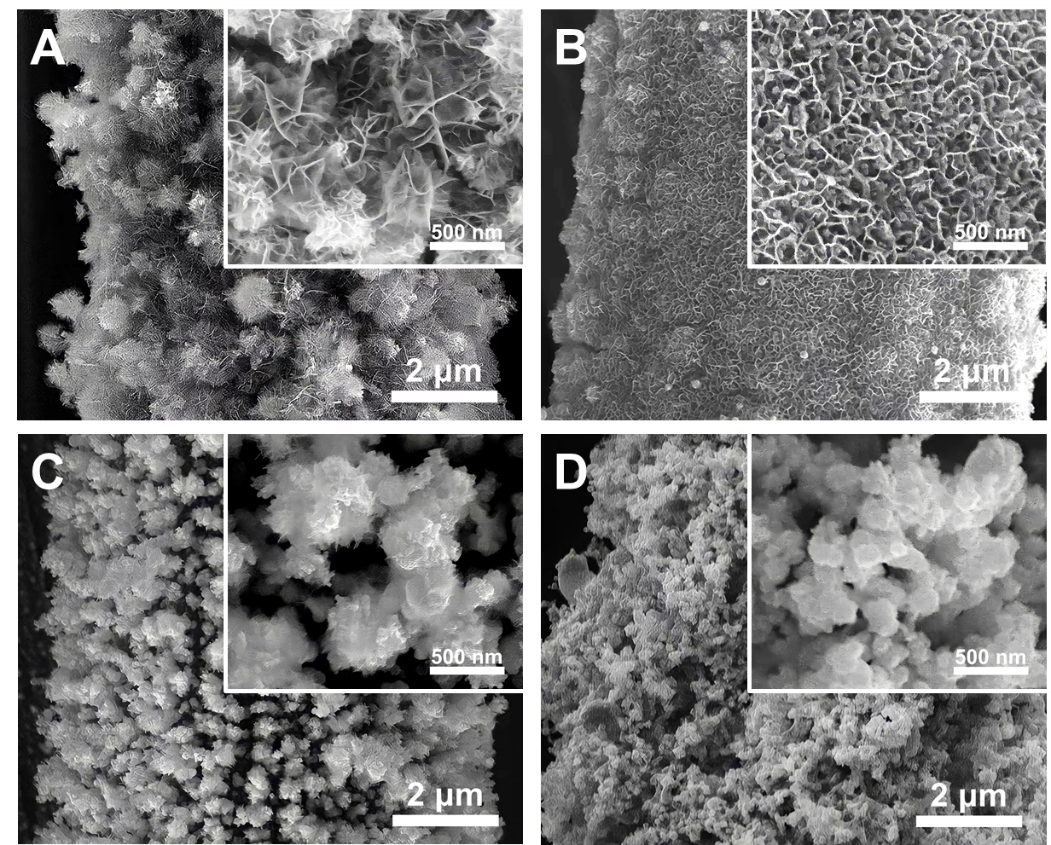


**Fig. S2.** SEM of Cu-Co-Ni-S/CP at different concentrations of CuCl_2_·2H_2_O solutions: (A) 0.5 mM; (B) 1 mM; (C) 2 mM; (D) 3 mM.


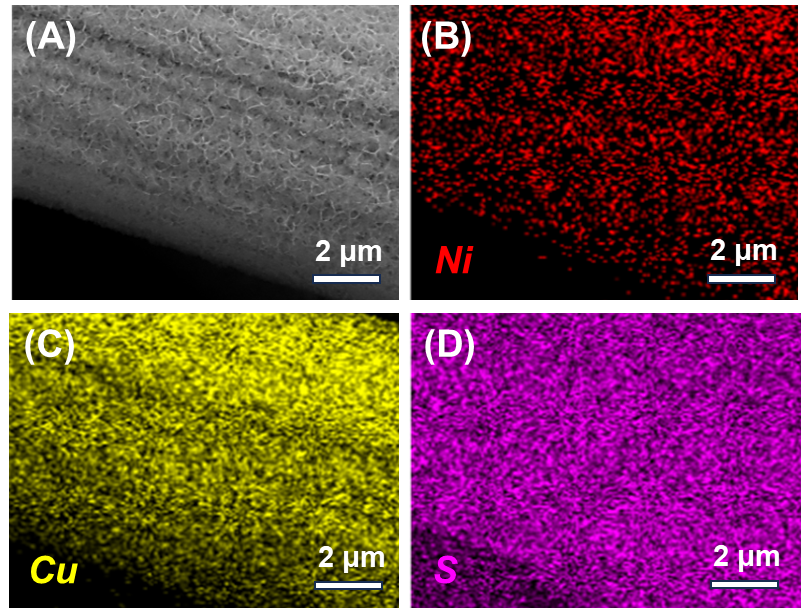


**Fig. S3.** Characterizations of Cu-Ni-S/CP nanostructure-based working electrode: **A** SEM image of Cu-Ni-S/CP; **B–D** corresponding elemental mapping images of Ni, Cu, and S.


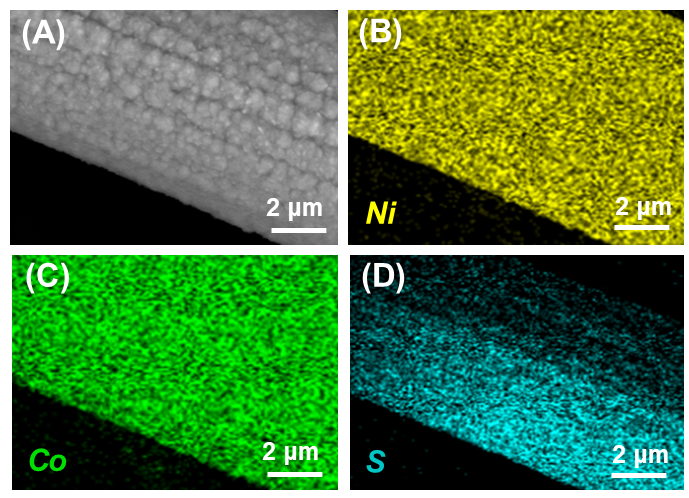


**Fig.S4.** Characterizations of Co-Ni-S/CP nanostructure-based working electrode: **A** SEM image of Cu-Ni-S/CP; **B–D** corresponding elemental mapping images of Ni, Cu, and S.


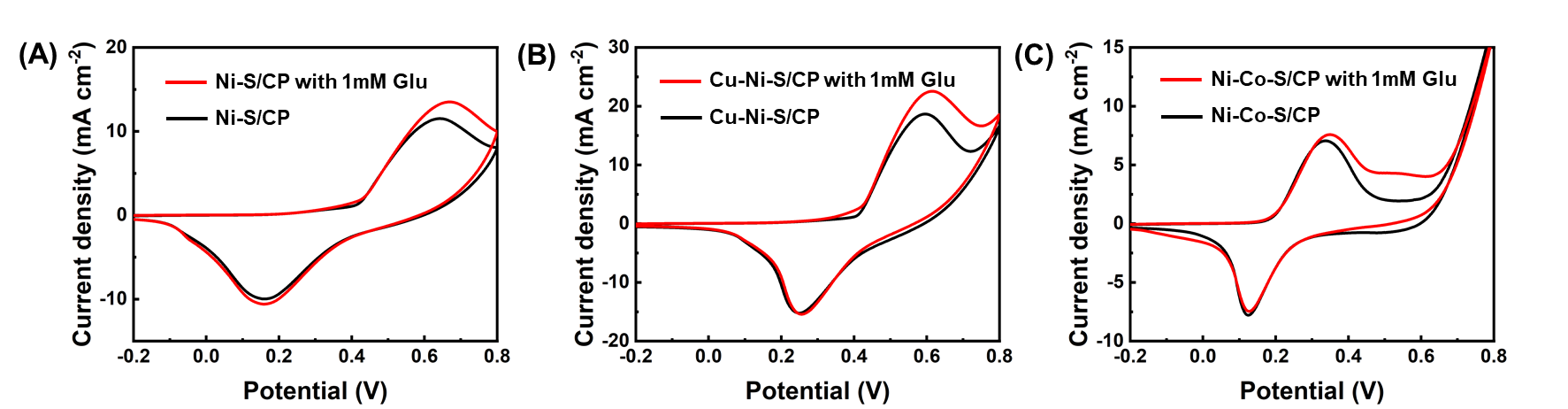


**Fig. S5.** CVs of **A** Ni-S/CP; **B** Cu-Ni-S/CP; **C** Ni-Co-S/CP in the absence and presence of 1 mM glucose in 0.15 M NaOH at the scan rate of 100 mV s^−1^.


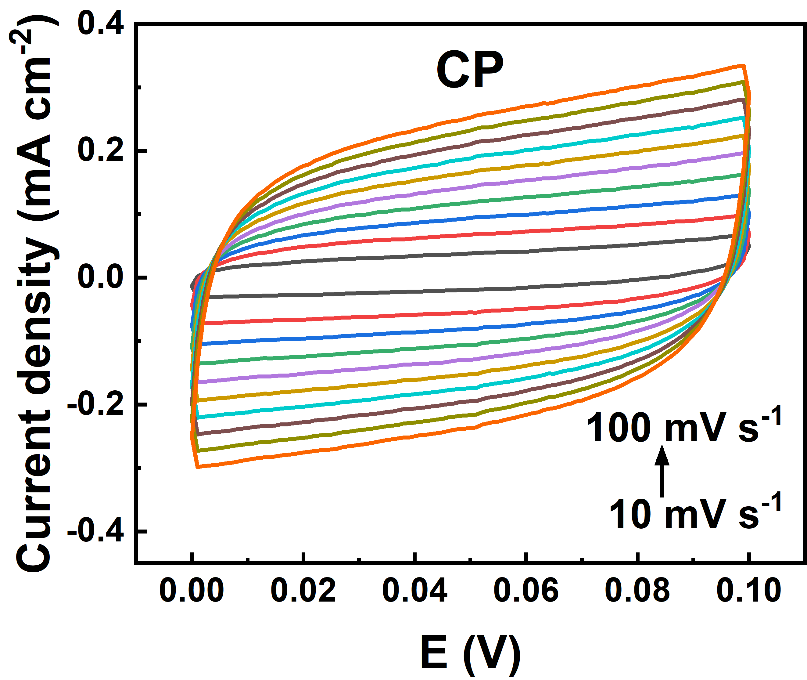


**Fig. S6.** CVs of CP electrodes measured in the applied potential range from 0.00 V to +0.10 V at various scan rates.


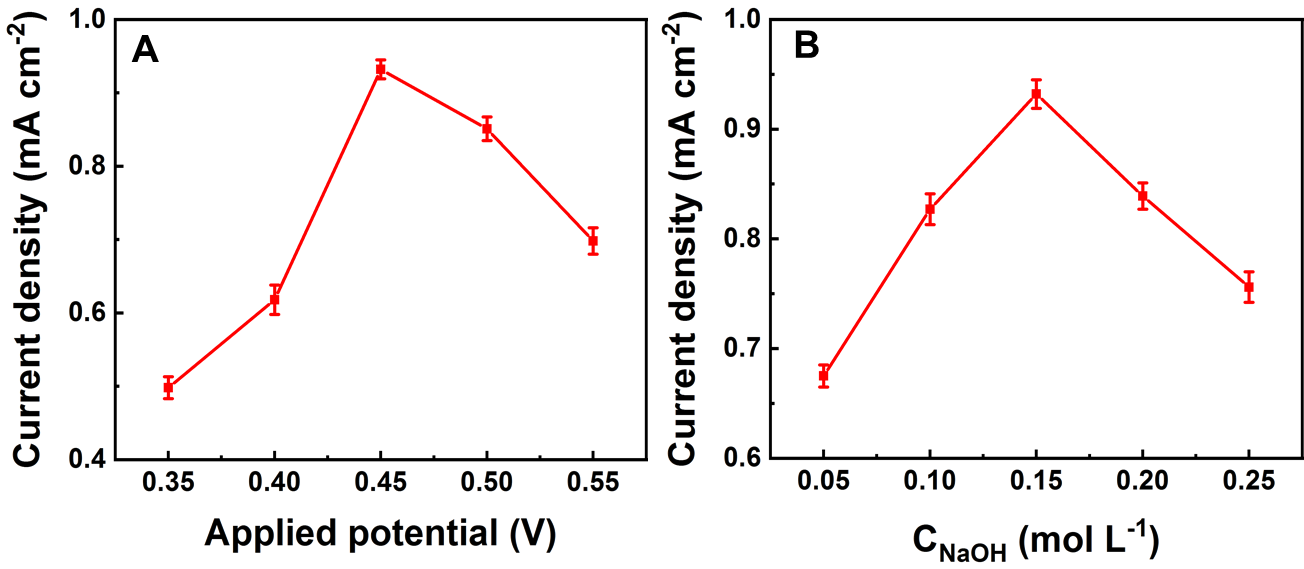


**Fig. S7.** Effects of experimental conditions on the current density response of 0.1 mM glucose: (A) Applied potential; (B) NaOH concentration.


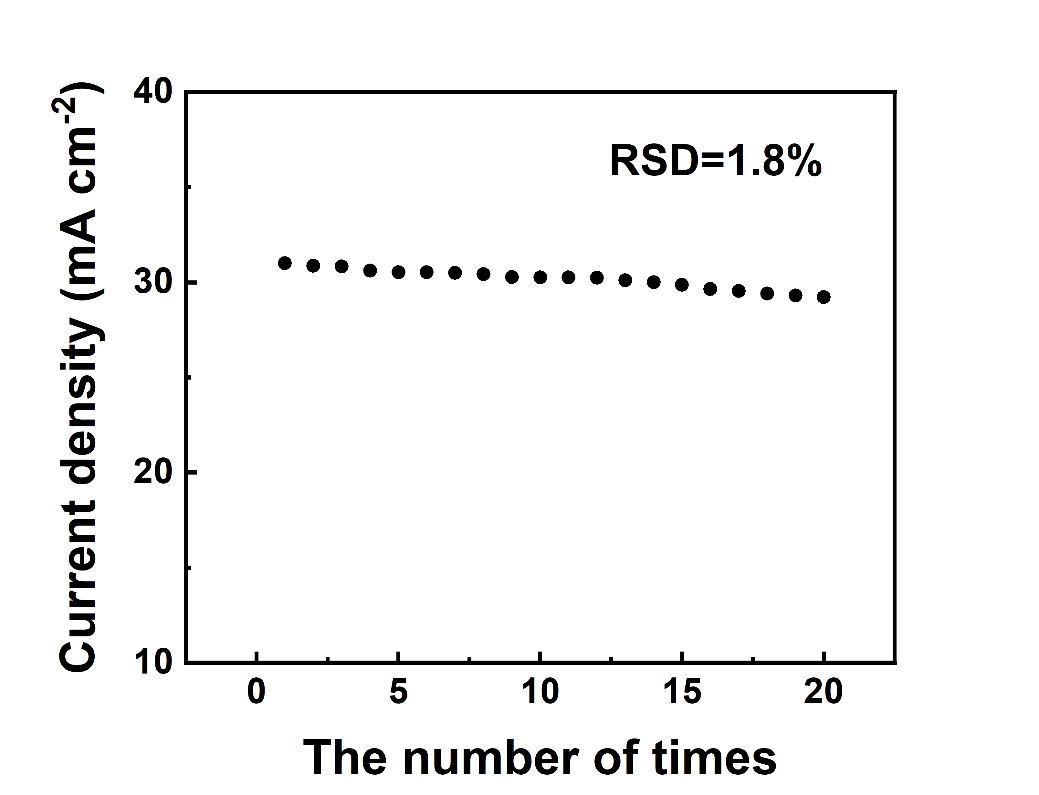


**Fig. S8.** The reusability test of Cu-Co-Ni-S/C electrode.
